# Analysis of thermal and dynamic mechanical properties of epoxy bio-composites reinforced with sisal fibers and carbon nanotubes

**DOI:** 10.1038/s41598-025-21546-w

**Published:** 2025-10-28

**Authors:** Dinesh Kumar Rao, Chandra Kant Kaithwas, Naman Jain, Udayashankar Shivaramakrishna, Jagadeesha Thimmaiah, Dayanand M. Goudar, Deesy G. Pinto, Subraya Krishna Bhat, Kandavalli Raju

**Affiliations:** 1 Department of Mechanical Engineering, Dr. Ram Manohar Lohia Avadh University, Ayodhya, 224 001 India; 2 Department of Mechanical Engineering, ABES Engineering College, Ghaziabad, 201 009 India; 3 Department of Mechanical Engineering, VTU, Belgaum, 590 018 India; 4 Department of Mechanical Engineering, National Institute of Technology, Kozhikode, 673 601 India; 5 Department of Mechanical Engineering, Tontadarya College of Engineering, Gadag, 582 101 India; 6 GeoBio Tec, Department of Civil Engineering & Architecture, University of Beira Interior, Calçada de Lameiro nº6, 6200-358 Covilhã, Portugal; 7 Department of Civil Engineering & Geology, University of Madeira, Campus da Penteada, 9020-105 Funchal, Portugal; 8 Department of Mechanical and Industrial Engineering, Manipal Institute of Technology, Manipal Academy of Higher Education, Manipal, 576104 Karnataka India; 9 Department of Mechanical Engineering, St. Joseph Engineering College, Mangaluru, 575 028 India

**Keywords:** Dynamic mechanical analysis, Bio-composite, Carbon nanotube, Glass transition temperature, Engineering, Materials science, Nanoscience and technology

## Abstract

The present study focuses on the fabrication and analysis of thermal and dynamic mechanical properties of epoxy bio-composites reinforced with 15 wt.% sisal fibers and varying carbon nanotube (CNT) content (0–2.0 wt.%). As per the results, incorporation of 1.0 wt.% CNT significantly enhances thermal and mechanical properties of the composite. Compared to the baseline composite without CNTs, thermal degradation onset has been improved by approximately 13%, while crystallinity and thermal resilience also increased. The storage modulus and loss modulus rose by approximately 79% and 197% respectively, indicating greater stiffness and energy absorption capacity. The damping factor (tan δ) decreased by over 56%, implying enhanced load-bearing capability with reduced energy dissipation. These improvements are attributed to better interfacial bonding and uniform CNT dispersion at 1.0 wt.%. The SEM analysis of epoxy bio-composites also revealed that the optimal dispersion and strong interfacial bonding are achieved at 1.0 wt.% CNT. Overall, the findings demonstrate that optimal thermal stability and viscoelastic properties occur at low CNT content in natural fiber composites, making them suitable for advanced structural applications in automotive, aerospace, and packaging sectors.

## Introduction

Because of their excellent strength-to-weight ratio, affordability, and eco-friendliness, natural fiber-reinforced polymer composites are getting more attention in modern engineering. Among these materials, sisal fiber is gaining more interest, due to its impressive tensile strength, stiffness, and widespread availability^1^. Natural fiber composites have a long history, with early examples including Egyptians reinforcing clay with straw around 4000 BC and Mesopotamians developing plywood around 3400 BC^2^. The Mongols also advanced in composite technology by creating bows from animal glue, wood, and bone^3^. These historical foundations paved the way for modern composite systems, which incorporate natural fibers such as sisal, coconut palm leaves, wood (composed of cellulose fibers in a lignin matrix), and animal bones (containing collagen fibers in an apatite matrix).w

The sisal fiber is one of the most widely used natural lignocellulosic fibers owing to its high tensile strength, low density, and biodegradability. It is abundantly available, cost-effective, and renewable, making it an attractive reinforcement for polymer composites. The fiber also possesses good thermal stability and strong interfacial bonding potential with thermosetting resins such as epoxy, which enhances the load-bearing capacity of the composites. Moreover, the use of sisal fiber supports sustainability by reducing reliance on synthetic fibers like glass, while simultaneously lowering the overall environmental footprint. These attributes make sisal fiber a suitable candidate for developing epoxy-based bio-composites for structural and tribological applications. The nineteenth and twentieth centuries saw major advancements, including the emergence of synthetic polymers like polyester and vinyl, and the introduction of glass-reinforced composites during World War II. These innovations led to industrial applications in boat hulls, electrical components, and automotive parts. Post-war manufacturing techniques, such as pultrusion, resin transfer molding, and vacuum bag molding expanded the composite usage^4,5^. The introduction of carbon fiber in 1961 revolutionized high-performance applications in aerospace, marine, and sports industries. By the 1990s, composites became recognized as lightweight and durable alternatives to metals and engineered polymers, partly due to the integration of carbon nanotubes (CNTs) which improved mechanical, thermal, and electrical properties.

Natural fiber-reinforced polymer composites have garnered significant attention due to their excellent strength-to-weight ratio, low cost, biodegradability, and sustainability^6,7^. A study on short sisal fiber-reinforced polypropylene (PP) composites highlighted temperature-dependent trends in storage modulus and loss modulus, revealing that treated fibers surpassed untreated ones due to better interfacial adhesion^8^. Additionally, research by Nair et al.^9^ indicated that sisal-polystyrene (PS) composites demonstrated improved thermal stability, with decomposition beginning at 329 °C for 20% sisal-PS blends, compared to 288 °C for pure PS. This enhancement can be attributed to the interactions between the fibers and the matrix. Dynamic mechanical analysis (DMA)^10^ of these composites revealed a reduced storage modulus with increasing temperature, which is connected to segmental mobility. While the presence of residual solvent in composites lowered their glass transition temperature (T_g_) relative to pure PS. Jayaraman^11^ optimized sisal-PP composites via hot-pressing and vacuum techniques. Findings show that 15–35% fiber mass fraction and more than 10 mm fiber length were critical for optimal mechanical properties. Research by Joseph et al.^12^ highlighted the degradation of sisal/PP composites when exposed to water and UV (Ultraviolet) determining their vulnerability. Specifically, moisture induced plasticization had weakened the fiber-matrix interfaces resulting in a decline in tensile properties. Rong et al.^13^ studied fiber treatment strategies to improve the sisal-epoxy adhesion while maintaining intercellular bonding, leading to enhanced tensile strength and laminate performance. Similarly, Sreekumar et al.^14^ achieved best flexural properties in sisal-polyester composites at 50% fiber loading and 30 mm length, although excess fiber content and length could lead to increased brittleness. Idicula et al.^15^ fabricated hybrid banana/sisal-polyester composite and reported that the optimal dynamic mechanical properties occur at 0.40 fiber volume fraction. The 3:1 ratio of banana to sisal fibers improved interfacial adhesion and stress transfer. Shahzad and Isaac^16^ compared hemp and glass fiber composites, noting that hemp exhibited brittle fatigue failure, unlike the progressive modulus decline observed in glass fiber, thus highlighting the distinctive failure mechanisms associated with natural fibers. Milanese et al.^17^ demonstrated that thermal treatment of sisal fibers in phenolic composites reduced variability in mechanical properties, recommending it for structural applications despite minimal strength changes. However, bio-composites based on natural fibers face critical challenges such as limited thermal stability, poor interfacial bonding between hydrophilic fibers and hydrophobic polymer matrices, and mechanical property degradation due to moisture absorption. Current research focuses on integrating CNTs into sisal-epoxy systems to enhance toughness, stiffness, and strength, leveraging CNTs’ nano-reinforcement potential^18^. Epoxy-based composites are valued for their adhesion, dimensional stability, and chemical resistance. Reinforcement with natural fibers such as sisal offers sustainability through low density, renewability, and biodegradability, though with limitations in thermal stability and strength. Incorporating carbon nanotubes (CNTs) overcomes these drawbacks by providing exceptional stiffness, strength, and improved thermal and tribological properties. The resulting sisal–CNT/epoxy bio-composites combine eco-friendliness with high performance, making them suitable for lightweight structural, automotive, aerospace, and wear-resistant applications^19–21^. This compilation of historical knowledge, empirical evidence, and advanced innovations highlights the capacity of sisal-CNT-epoxy composites to meet global needs for sustainable, high-performance materials, merging traditional material knowledge with contemporary nano-technology.

Sathish et al.^21^ explored the integration of carbon nanotubes (CNTs) into sisal and jute fiber composites to enhance their mechanical and thermal properties. Incorporating up to 7% CNTs led to a significant improvement in tensile strength by 63.9%, flexural strength by 46.6%, and impact resistance by 58.3%. The study suggests that such CNT-reinforced natural fiber composites hold promise for applications requiring lightweight and thermally stable materials, such as in automotive and construction industries. Pantano et al.^22^ investigated the incorporation of low fractions of carbon nanotubes (CNTs) into short sisal fiber-reinforced epoxy composites. By optimizing the dispersion of CNTs within the epoxy matrix, the researchers achieved significant improvements in both static and fatigue strength of the bio-composites. Rodríguez-González et al.^23^ explored the effects of spray coating graphene nano-platelets onto sisal fibers used in bio-composite laminates. The treatment led to notable enhancements in the electromechanical properties of the composites, including increased electrical conductivity and mechanical strength. Kamesh et al.^24^ integrated graphene into sisal/glass fiber-reinforced polymer composites. The addition of graphene resulted in improved mechanical properties, reduced water absorption, enhanced flame retardancy, and decreased swelling behavior. CNTs and graphene are effective in significantly improving the mechanical and thermal properties of polymer composites through nanoscale reinforcement. For instance, the use of CNTs and graphene in hybrid natural fiber-reinforced systems has demonstrated increased modulus, strength, and thermal resistance, attributed to improved load transfer and interfacial interaction^25–27^.

Due to high tensile strength, stiffness, and abundant availability sisal fiber is a promising reinforcement material. But there are other challenges such as limited thermal stability, poor interfacial bonding between hydrophilic fibers and hydrophobic polymer matrices, and mechanical property degradation due to moisture absorption. To address these limitations, carbon nanotubes (CNTs) have emerged as effective nano-reinforcements due to their exceptional mechanical strength, thermal conductivity, and aspect ratio. Incorporation of CNTs into sisal-polymer composites can significantly enhance stiffness, toughness, and thermal performance. However, CNT agglomeration and poor dispersion remain a major bottleneck, which can deteriorate composite’s performance.

The addition of CNTs to fiber-reinforced epoxy composites has been widely reported to enhance thermal conductivity due to their extremely high intrinsic values, ranging from 3,000 to 3,500 W/m·K^28^. Even at low concentrations, CNTs form conductive networks that facilitate heat transfer, with aligned CNT–epoxy composites showing significantly higher conductivity compared to random orientation^29^. Molecular dynamics simulations and micromechanical models confirm that CNT reinforcement not only enhances thermal conductivity but also reduces thermal expansion in hybrid fiber composites^30,31^. Experimental studies further demonstrate that hybrid CNT/fiber systems achieve better thermal performance than single-filler composites^32^.

This study aims to fabricate and characterize hybrid epoxy-based bio-composites reinforced with sisal fibers (15 wt.%) and varied CNT content (0–2 wt.%). The primary objective is to investigate how CNT loading influences the thermal stability, glass transition behavior, and viscoelastic properties of the composites. The work applies differential scanning calorimetry (DSC), thermogravimetric analysis (TGA), and dynamic mechanical analysis (DMA) to evaluate these effects.

## Materials and methods

The bio-composites enhanced with sisal fibers were sourced from the Women Development Organization in Dehradun, Uttarakhand, India. The continuous phase of the composites was made up of epoxy resin (LY556) that was cured with its hardener (HY951). To improve interfacial bonding sisal fibers were subjected to a 5 wt.% NaOH (sodium hydroxide) solution at room temperature for 4 h, followed by thorough washing with distilled water until neutral pH was achieved. The fibers were then oven-dried at 80 °C for 24 h to eliminate moisture content. Then sisal fibers were manually cut to an average length of approximately 30 mm, which aligns with previous studies recommending the same length for optimal mechanical performance^11^. Multi-walled carbon nanotubes (MWCNTs) used had an average outer diameter of ~ 20–30 nm and length ranging between 1–10 µm, as specified by the supplier (Adnano Technologies Pvt. Ltd., India).

**Fig. 1:**
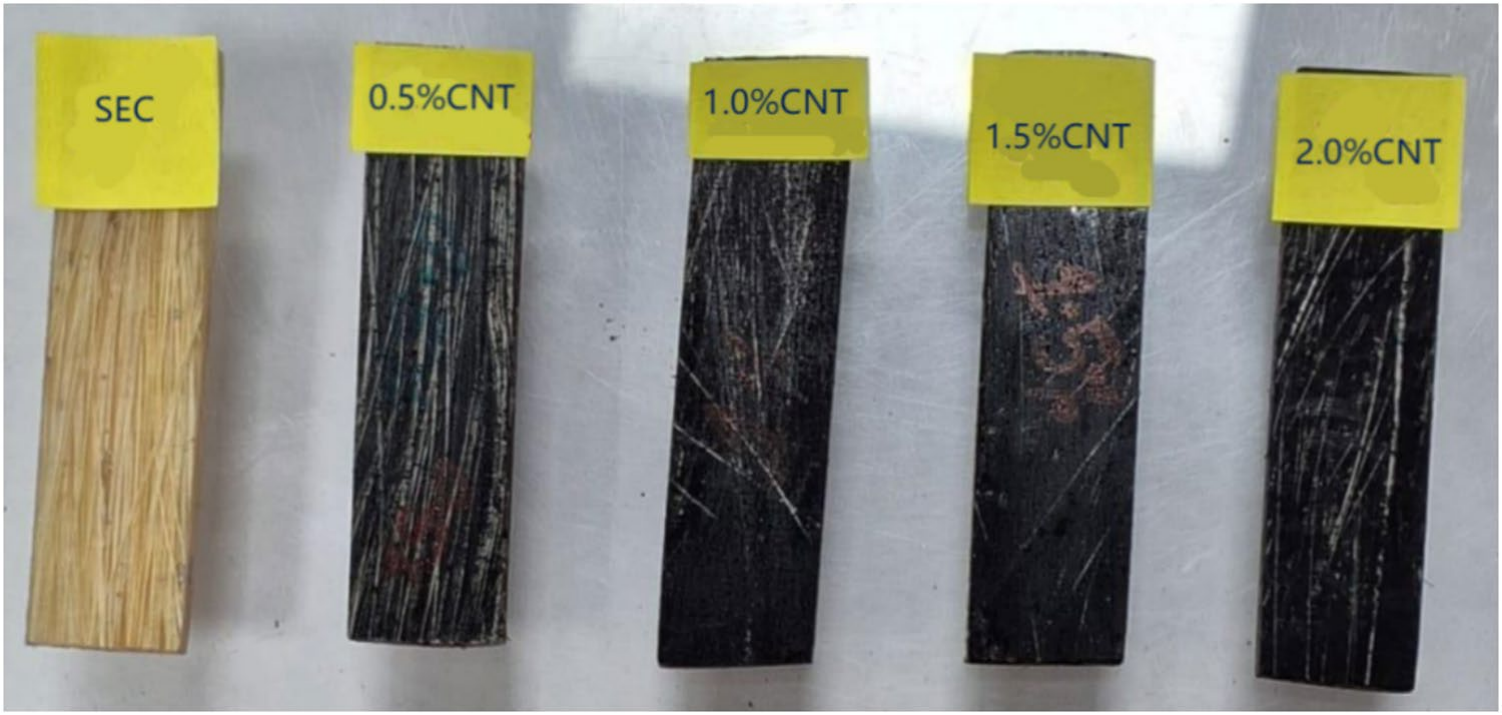
Fabricated bio-composite specimens.

The fabrication of composite involved a hand lay-up method^12^ utilizing a stainless steel mold (300 mm × 200 mm × 3 mm). A wax-based releasing agent was applied to the mold’s surface to assist in demolding. Unidirectional sisal fibers, maintained at 15 wt.% of the bio-composite, were evenly distributed within the mold. For uniform dispersion of CNTs, the desired amount of CNTs was first ultra-sonicated in the epoxy resin using a probe sonicator (400 W, 20 kHz) for 30 min. This step was followed by mechanical stirring at 500 rpm for an additional 15 min to ensure better distribution. The hardener was then added in the ratio of 1:10 wt.% to the mixture before the hand lay-up. These steps were taken to reduce agglomeration and ensure better interfacial bonding. Two flat plates were used to compress the stacked layers under a 50 kg load to remove air voids, ensure flatness of the sheet, and promote uniform curing. The setup was allowed to cure at room temperature for 24 h. After curing, the bio-composite sheet was carefully removed from the mold, resulting in a 3 mm thick panel. Specimens were labeled according to their CNTs content (Table 1 & Fig. 1). This technique guaranteed consistent fiber alignment, controlled dispersion of CNTs, and uniform distribution of the matrix, adhering to standardized dimensions and weight fractions.

**Table 1:** Nomenclature of bio-composites.

Nomenclature	Specification of bio-composites
SEC	Sisal fiber (constant 15 wt.% loading) epoxy composite
SEC + 0.5% CNT	Sisal fiber (constant 15 wt.% loading) epoxy composite with 0.5 wt.% CNT additive
SEC + 1.0% CNT	Sisal fiber (constant 15 wt.% loading) epoxy composite with 1.0 wt.% CNT additive
SEC + 1.5% CNT	Sisal fiber (constant 15 wt.% loading) epoxy composite with 1.5 wt.% CNT additive
SEC + 2.0% CNT	Sisal fiber (constant 15 wt.% loading) epoxy composite with 2.0 wt.% CNT additive

## Characterization and testing techniques

### Thermogravimetric analysis (TGA)

The Thermogravimetric analysis (TGA) is employed for evaluating the thermal stability and degradation characteristics of composites. By employing TGA, one can gain comprehensive insights into their thermal properties. TGA, STA 409 Netzsch was employed to conduct TGA analysis. For analyzing the thermal stability of each composite, approximately 2–8 mg samples were heated from 30ºC to 750ºC at a heating rate of 10ºC/min in an inert gas (nitrogen) environment^12^. Amount of weight loss with respect to temperature is noted for examining the thermal stability and decomposition behavior of composites.

### Differential scanning calorimetry (DSC)

An advanced thermal analysis technique used to examine the thermal transition of different materials was studied using Differential Scanning Calorimeter (DSC) (Hitachi model DSC 600). This encompasses the glass transition temperature (T_g_), melting temperature (T_m_), and crystallization behavior. The experiments were carried out in the temperature range of 30ºC –400ºC at a heat flow rate of 10ºC/min under nitrogen atmosphere purged at 20 ml/min. Nitrogen was used for efficient heat transfer and removal of volatiles from the samples.

### Dynamic mechanical analysis (DMA)

The Dynamic mechanical analysis (DMA) was carried out to assess the viscoelastic behavior of the sisal/CNTs epoxy based composite using a Hitachi DMA7100 analyzer, adhering to the ASTM D5026 standard^33^. The experiments were executed in sinusoidal tensile mode with rectangular samples measuring 20 mm × 10 mm. The temperature ranging from 25 °C to 180 °C was applied, with a heating rate of 2 °C/min. It is important to note that the observed ‘T_g_’ and modulus values could be influenced by residual moisture in the sisal fibers, which are inherently hygroscopic. Since no variation in pre-conditioning was applied across samples, the reported thermal–mechanical results reflect the performance under ambient moisture content.

## Results and discussion

### TGA thermograms of bio-composites

**Fig. 2:**
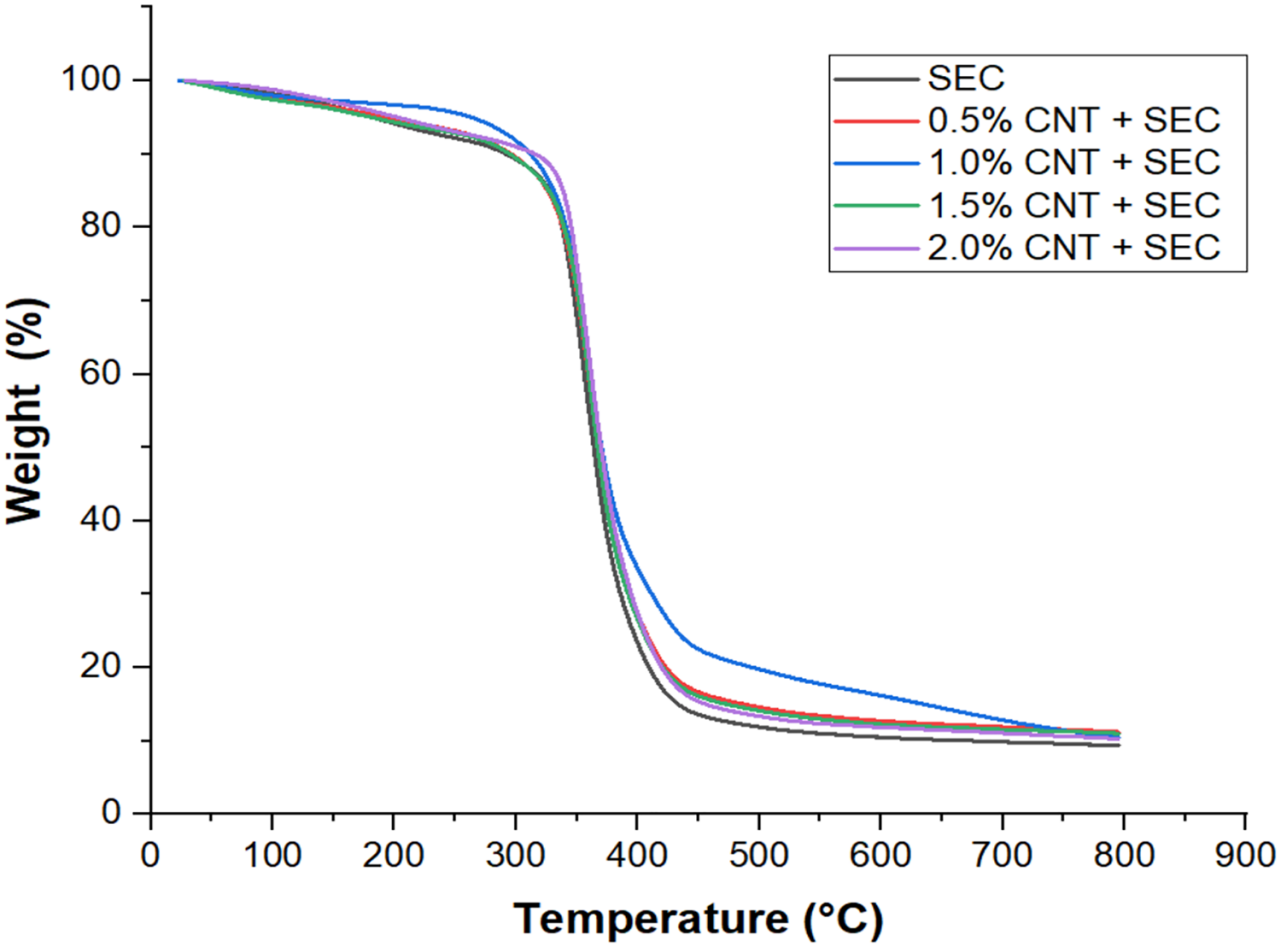
TGA thermograms of bio-composites.

The Fig. 2 illustrates the thermograms of nano-cellulose and epoxy bio-composites. Additionally, Table 2 summarizes the percentage of weight loss at various temperatures. The bio-composites exhibit three primary degradation stages, as illustrated in Fig. 2. The thermal degradation of sisal with epoxy and all composites occurred within the temperature range of 30ºC-100ºC during the initial stage of low temperature degradation. This loss may be attributed to the desorption of volatile impurities, residual solvents, or the initial stages of hemicellulose decomposition, which can begin degrading above 100 °C under inert conditions^34^. The Fig. 2 shows the variation of weight loss of sisal epoxy composites at various temperatures. It has been observed that as the weight percentage of CNT increases the percentage of weight loss decreases up to 1.5% CNT; however, beyond 1.5% CNT, the percentage of weight loss begins to increase again^35^. So, at 30ºC-100ºC weight loss % is minimum at 1.5% CNT i.e. 1.23% and maximum at 2% CNT i.e. 2.58%. As the temperature is increased, it has been further observed that the minimum weight loss of the bio-composites is at 1% of CNT addition. So, the maximum stability is achieved at 1% CNT + SEC. The maximum thermal stability observed at 1.0 wt.% CNT + SEC can be attributed to the optimal dispersion of carbon nanotubes within the epoxy matrix^36^. At this concentration, CNTs are sufficiently distributed to form an effective thermal barrier and reinforce the polymer network, thereby delaying the onset of degradation. Their high aspect ratio and intrinsic thermal conductivity enhance heat dissipation and restrict polymer chain mobility, increasing thermal resistance. Furthermore, strong interfacial interactions between CNTs, the epoxy matrix, and sisal fibers contribute to the improved structural integrity and reduced mass loss.

**Table 2:** TGA results of bio-composites.

Temperature (°C)	Weight loss (%) of bio-composites				
	SEC	0.5% CNT + SEC	1.0% CNT + SEC	1.5% CNT + SEC	2.0% CNT + SEC
30–100	1.80 ± 0.05*	2.44 ± 0.06	2.01 ± 0.05	1.23 ± 0.04	2.58 ± 0.06
100–380	66.14 ± 0.35	61.71 ± 0.31	57.62 ± 0.28	60.13 ± 0.30	62.85 ± 0.33
380–420	82.80 ± 0.40	79.23 ± 0.38	72.28 ± 0.35	80.04 ± 0.39	79.87 ± 0.36
420–790	90.60 ± 0.42	88.72 ± 0.39	89.38 ± 0.38	89.65 ± 0.40	88.93 ± 0.37

* Standard deviation.

### DSC thermograms of bio-composites

**Fig. 3:**
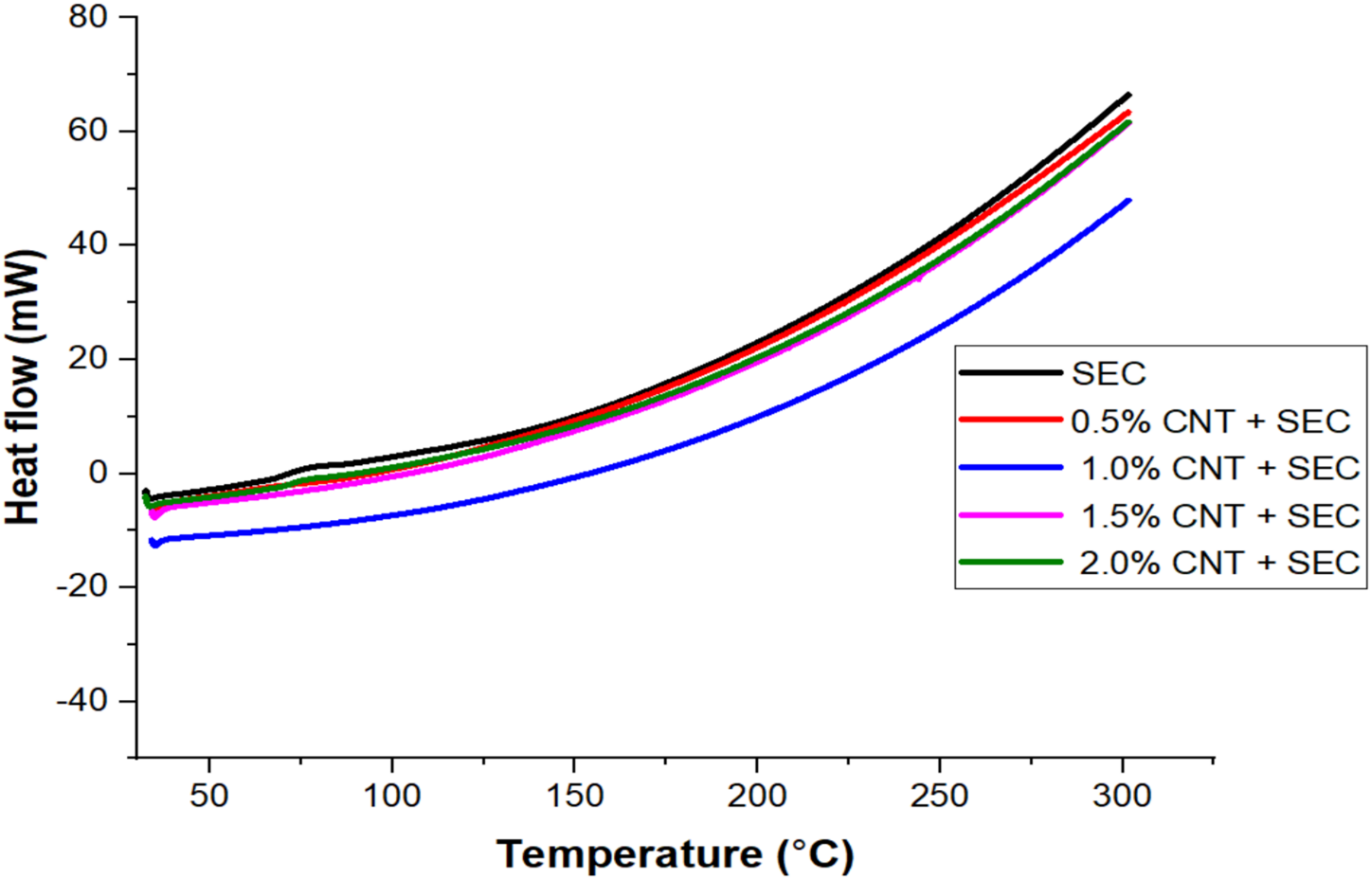
DSC thermograms of bio-composites.

The DSC thermograms of epoxy composites reinforced with 15 wt.% sisal fiber and varying quantities of carbon nanotubes (CNTs) (0.5, 1.0, 1.5, and 2.0 wt.%) are depicted in Fig. 3. Two distinct endothermic alterations are observed in all the five thermograms as the temperature is increased. The initial endothermic maxima at 110 °C were observed in all sisal fibers and CNT as a result of the moisture loss^37^. TGA studies have previously documented this form of moisture loss in response to an increase in temperature. The higher heat absorption observed in the 1.0 wt.% CNT + SEC composite during initial endothermic transition can be attributed to the improved dispersion of CNTs, which enhances interfacial bonding and encapsulation of the sisal fibers. Sisal fibers contain hydrophilic constituents—cellulose, hemicellulose, and lignin that readily absorb atmospheric moisture. When CNTs are well dispersed (as in the 1.0 wt.% sample), they contribute to better matrix penetration and microstructural uniformity, enabling more effective interaction with the fibers’ hydrophilic groups. As a result, more bound moisture is retained within the interface and requires greater heat input for evaporation^38^. In contrast, at lower CNT content, the interaction is weaker, while at higher CNT loading (1.5% and 2.0%), CNT agglomeration can lead to voids and poor wetting, reducing the quantity of absorbed water and the associated heat absorption. A distinct endothermic nature was observed in each of them as a result of a change in the composition of CNT. The final decomposition of CNT and fibers was observed at 380ºC-390 °C, which may be attributed to the decomposition of the fibers’ constituents. The thermal stability of hemp fiber was improved by the addition of 1.0 wt. % of CNT^34^. A superior reinforcement for polymeric films and composites that are intended for use in industrial applications can be provided on the basis of the enhanced thermal stability of 1.0 wt. % of CNT, which is the consequence of an increase in the crystallinity of the material.

### Dynamic mechanical properties of bio-composites

The Table 3 summarizes the dynamic mechanical properties of the epoxy-sisal-CNT bio-composites. A clear trend is observed in the storage modulus (${E}{\prime}$), which increases with CNT addition up to 1.0 wt.% (3859.28 MPa) and then decreases beyond that concentration. This trend suggests that well-dispersed CNTs enhance matrix stiffness, likely due to better stress transfer and reduced polymer chain mobility. At higher CNT contents (1.5% and 2.0%), possible agglomeration reduces the reinforcing efficiency. The loss modulus (${E}^{{\prime}{\prime}}$) follows a similar trend, peaking at 1.0 wt.% CNT (539.44 MPa), indicating superior energy absorption capacity under cyclic loading. This peak is associated with enhanced interfacial friction and mechanical damping due to well-dispersed CNTs. The damping factor (tan δ) exhibits the opposite behavior, highest in the control sample (0.58) and lowest in the 1.0 wt.% CNT composite (0.25), indicating reduced energy dissipation and increased elastic response, which reflects stronger interfacial bonding and restricted molecular motion. The glass transition temperature (T_g_) also increases with CNT content, reaching a maximum of 89.26 °C at 1.0 wt.% CNT, supporting the claim of enhanced thermal resistance. This rise in ‘T_g_’ is attributed to the restriction of segmental mobility due to improved matrix–fiber–CNT interaction.

**Table 3:** Dynamic mechanical properties of bio-composites.

Bio-composites	Storage modulus ${E}{\prime}$(MPa)	Loss modulus $E"$(MPa)	Damping factor ‘tan δ’	T_g_ (°C) at loss modulus	${E}{\prime}$ at 120 °C (MPa)	Crosslink Density ‘νₑ’ (10^3^ mol m^-3^)	Bound polymer fraction (${f}_{b}$)
SEC	2159.37	181.63	0.58	80.16	230.77	23.5	
0.5% CNT + SEC	3165.60	294.34	0.26	85.68	286.44	29.2	0.55
1.0% CNT + SEC	3859.28	539.44	0.25	89.26	156.49	16.0	0.57
1.5% CNT + SEC	2773.18	251.35	0.28	86.34	175.07	17.9	0.52
2.0% CNT + SEC	2306.484	221.01	0.35	73.18	119.36	12.2	0.39

#### Storage modulus

**Fig. 4:**
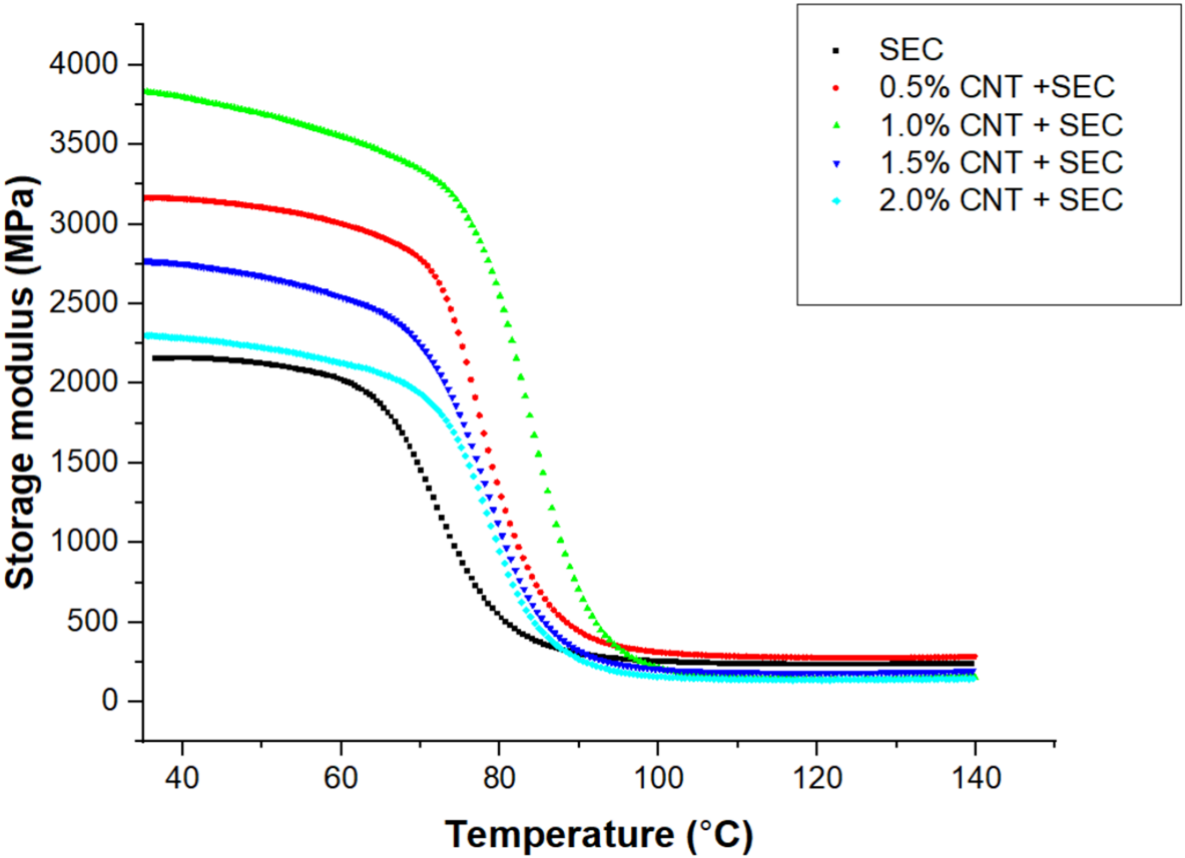
Storage modulus curves of bio-composites.

The storage or elastic modulus is used to assess the rigidity of a polymer composite. The Fig. 4 illustrates the temperature-dependent variations in storage modulus of epoxy composites reinforced with 15 wt.% sisal fiber and varying quantities of carbon nanotubes (CNTs) (0.5, 1.0, 1.5, and 2.0 wt.%) at a frequency of 1 Hz. The incorporation of rigid CNTs resulted in an improvement in the values of the storage modulus (${E}{\prime}$), indicating enhanced stiffness of the composite due to effective stress transfer and uniform CNT dispersion. In comparison to the epoxy matrix, each composite exhibited a higher value, with the composites containing 1.0 wt.% of CNT exhibiting the highest value. The composites containing 1.0 wt.% of CNT provided the most advantageous value in the vitreous region^38^. CNTs act as nanoscale bridges between the hydrophilic sisal fibers and the hydrophobic epoxy matrix. Their high surface area allows strong Van der Waals forces and potential covalent interactions with both the fiber and matrix. This results in tighter bonding at the interface and reduced interfacial defects, which typically weaken the composite performance. This outcome may be attributed to the inclusion of nano-cellulose, which enhances the composite’s stiffness, thereby increasing its value. Hydrogen bonds between the polar groups of the epoxy matrix and the sisal fiber with 1.0 wt.% CNT may also be responsible for the enhanced value, which is achieved through homogeneous distribution. In contrast, the epoxy matrix exhibited the lowest value due to its minimal degree of stiffness. The value of composites containing more than 1.0 wt.% of CNT may have decreased due to agglomeration. The values were observed to diminish for epoxy and all sisal and CNT bio-composites as the temperature increased. The storage modulus of short sisal composites is illustrated in Fig. 4 as a function of temperature. Upon comparing the various composites, it is discovered that the value of ${E}{\prime}$ increases as the amount of CNT additive increases up to 1% and then decreases. The maximum and lowest values of ${E}{\prime}$ are 3859.28 MPa and 2159.37 MPa for composites 1.0% CNT + SEC and SEC respectively, in the vitreous region.

The strength-to-weight ratio (specific modulus) is an important parameter for evaluating the lightweight performance of structural materials. In this study, the storage modulus values from the DMA results (Table 3) were normalized by the composite density to estimate specific modulus. For the 1.0 wt.% CNT composite, a peak storage modulus of 3859.28 MPa was achieved. Given the average density of the sisal fiber-epoxy-CNT composite (~ 1.25 g/cm^3^)^36^, the estimated strength-to-weight ratio is approximately 3.09 MPa·cm^3^/g. This represents a notable improvement over the control (SEC) composite, which showed a specific modulus of 1.73 MPa·cm^3^/g. The enhancement is attributed to both the reinforcing effect of CNTs and improved fiber–matrix interaction. These results reinforce the claim that natural fiber composites, particularly when hybridized with CNTs, provide favorable mechanical performance at reduced weight, making them ideal for applications where lightweight design is critical, such as automotive or aerospace components.

#### Loss modulus

**Fig. 5:**
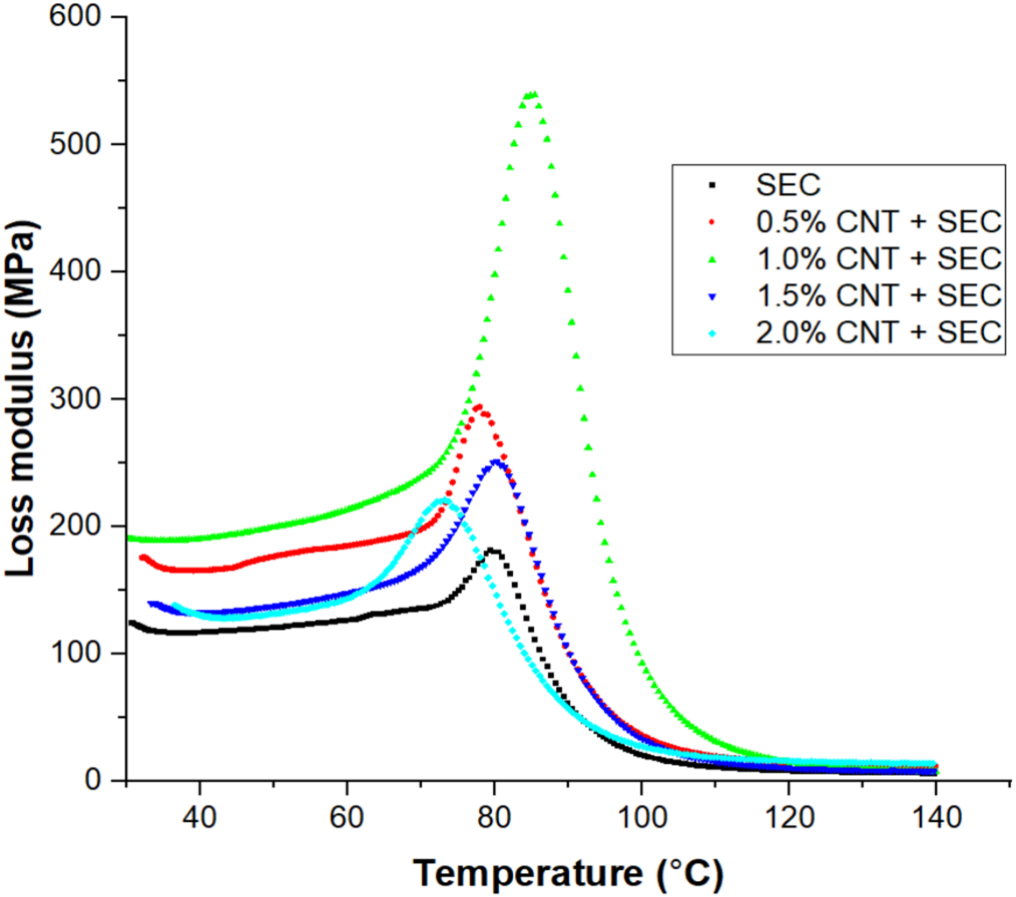
Loss modulus curves of bio-composites.

The loss modulus is defined as the amount of energy that is lost during each cycle of sinusoidal stress and is taken into consideration to be a viscous response of polymeric materials. A frequency of 1 Hz is used to depict the loss modulus vs temperature curve of epoxy sisal fiber and carbon nanotube (CNT) bio-composites, which can be found in Fig. 5. It is easy to determine the value of ‘T_g_’, which is the temperature that is linked with the highest apex of the loss modulus curve, because the loss modulus presents this information. The uppermost peak values of loss modulus for sisal fiber and CNT-reinforced epoxy bio-composites are summarized in Table 3, which contains a summary of the values that were measured. It is evident from Fig. 5 that the loss modulus gradually increases and reaches a peak value and then subsequently decreases as the temperature increases for all composites. The peak value occurs between 73 °C and 89 °C for all the composites. The curve’s highest peak was observed in the 1.0 wt.% of CNT with sisal fiber composite, while the lowest peak was observed in the 2.0 wt.% CNT with sisal fiber (15 wt.%) composite. The composite containing CNT at a concentration of 1.0 wt.% exhibited a shift in the peak toward a higher temperature (89.26 °C) in comparison to SEC and is 10.2% higher than that of SEC^39^.

#### Damping factor (tan δ)

**Fig. 6:**
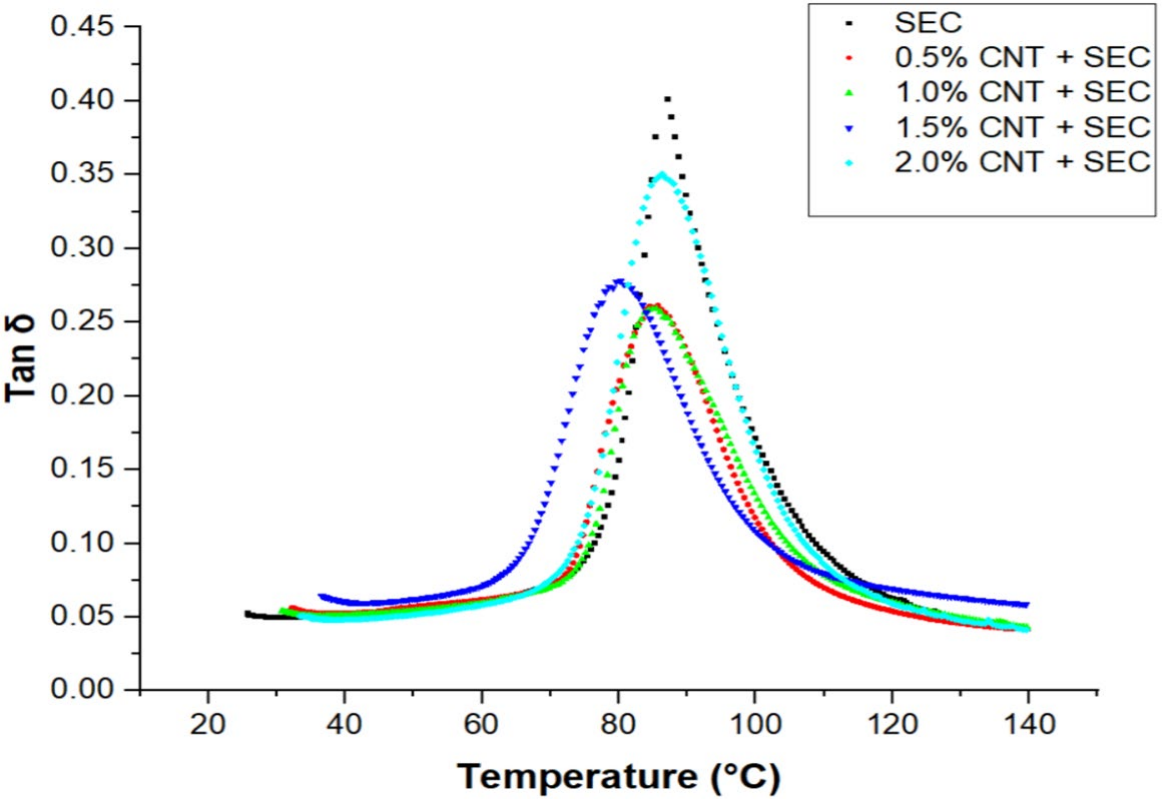
Damping factor curves of bio-composites.

The damping factor (tan δ) is the ratio of the loss modulus to the storage modulus or the ratio of the energy lost to the energy stored per oscillation cycle. The Fig. 6 illustrates the relationship between the temperature of the epoxy matrix and nano-cellulose bio-composites and the variation in ‘tan δ’ at a frequency of 1 Hz. The ‘tan δ’ curve is also beneficial in revealing the value of ‘T_g_’, similar to the loss modulus curve. Table 3 summarizes the measured values of the highest peaks in ‘tan δ’ curves of epoxy and nano-cellulose bio-composites. The curves increased until they reached their highest peak, at which point they began to decline. The sisal fiber-reinforced epoxy composite exhibited the highest apex, suggesting a greater degree of molecular mobility. The composite containing 1.0 wt. % of CNT and sisal fiber exhibited the lowest value, while the remaining composites exhibited intermediate values. The lowest ‘tan δ’ peak demonstrates low damping properties and a high load bearing capacity, and the converse is also true.

Dynamic mechanical analysis (DMA) of SEC/CNT composites reveals critical structural insights beyond basic ‘T_g_’ evaluation. Secondary relaxation is not clearly indicated in storage modulus but slightly appearing in ‘tan δ’ and E″curves (β at ~ 20 °C–30 °C) attributed to cellulose side-chain motion, which shift and reduce in intensity with CNT addition—indicating restricted polymer mobility and interphase formation. The primary α-transition (T_g_) increases from ~ 80 °C (SEC) to ~ 89 °C at 1.0 wt.% CNT, suggesting enhanced network stiffness. Crosslink density (νₑ) as per Eq. (1), calculated using storage modulus at 120 °C, peaks at 29,220 mol/m^3^ for 0.5% CNT, confirming CNT-induced network densification, while a decrease at ≥ 1.5% CNT indicates potential filler agglomeration. The bound polymer fraction ${(f}_{b})$ as calculated by Eq. (2) is highest at 1.0% CNT, highlighting significant polymer immobilization at the interface and stronger adhesion. However, at 2.0% CNT, both ‘νₑ’ and ${\prime}{f}_{b}{\prime}$ decrease, consistent with clustering. Together, these results confirm that CNTs improve composite stiffness and interfacial interaction at low loading, but excess CNTs hinder performance. These additional structural parameters validate the reinforcement mechanism and complement the mechanical property trends observed.

1 $${\nu }_{e}=\frac{{E}{\prime}}{3RT}$$

2 $${f}_{b}=1-\frac{{\text{tan }\delta }_{sample}}{tan{ \delta }_{matrix}}$$

where,

${\upnu }_{e}$ = Crosslink density (mol m⁻^3^).

E′ = Storage modulus in the rubbery plateau at 120ºC (Pa or N/m^2^).

R = Universal gas constant (8.314 J mol⁻^1^ K⁻^1^).

T = Absolute temperature (in Kelvin, K).

${f}_{b}$ = Fraction of bound or immobilized polymer.

tan δ_sample_ = Damping factor (peak value) of the CNT reinforced composite.

tan δ_matrix_ = Damping factor (peak value) of the SEC.

#### Surface morphology of bio-composites

**Fig. 7:**
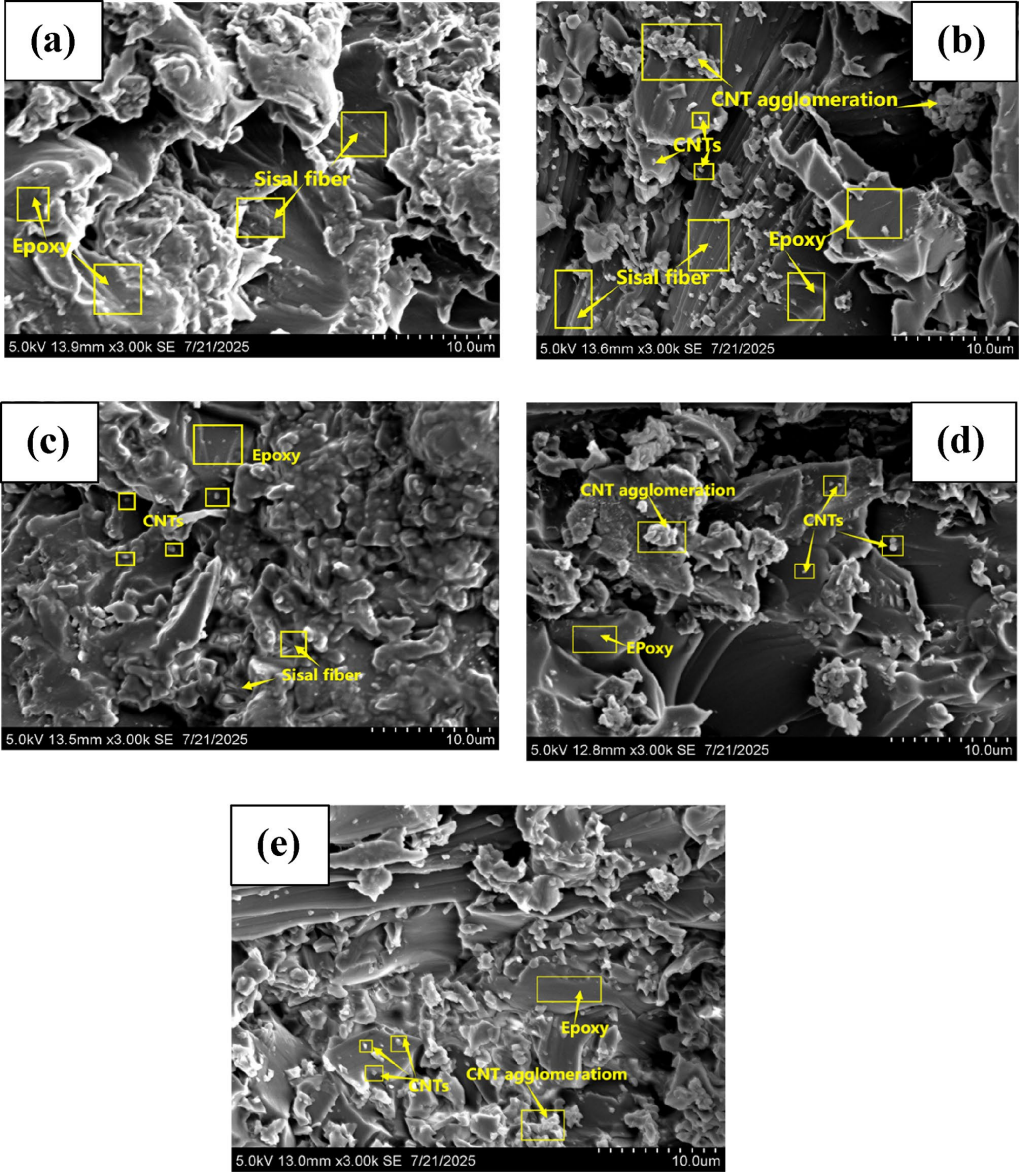
SEM micrographs of (**a**) SEC, (**b**) 0.5% CNT + SEC, (**c**) 1.0% CNT + SEC, (**d**) 1.5% CNT + SEC, and (**e**) 2.0% CNT + SEC.

The SEM micrographs of bio-composites are depicted in Fig. 7. The SEM analysis was conducted to evaluate the dispersion of carbon nanotubes (CNTs) and the interfacial bonding between sisal fibers and the epoxy matrix in the bio-composites. The baseline composite (SEC) (Fig. 7a) exhibited noticeable fiber pull-out and interfacial voids, indicating weak fiber–matrix adhesion. With the addition of 0.5 wt.% CNTs (Fig. 7b), partial improvement in the surface morphology was observed, with reduced voids and better fiber encapsulation. At 1.0 wt.% CNT loading, the SEM micrograph revealed uniform CNT dispersion and significantly enhanced fiber–matrix bonding, with minimal pull-out and a denser matrix structure (Fig. 7c). This suggests effective stress transfer and improved mechanical integrity at this CNT concentration. As CNT content increased to 1.5 wt.% (Fig. 7d) and 2.0 wt.% (Fig. 7e), agglomeration became apparent. Clusters of CNTs disrupted the matrix continuity and created micro voids, which can act as stress concentrators.

These morphological features potentially explain the observed decline in mechanical and thermal performance beyond 1.0 wt.% CNT addition. Overall, the SEM micrographs confirm that optimal dispersion and strong interfacial bonding are achieved at 1.0 wt.% CNT, correlating with the peak in thermal stability and viscoelastic properties. This underscores the importance of controlled CNT loading to enhance composite performance while avoiding agglomeration related defects. The SEM observations provide direct evidence of the morphological changes in sisal/epoxy composites with the addition of CNTs. The features such as reduced fiber pull-out, improved wetting of fibers by the matrix, and a uniform distribution of CNTs indicate enhanced interfacial bonding.

The enhanced interfacial adhesion in epoxy/sisal/CNT bio-composites can be attributed to multiple factors. The hydroxyl groups present in sisal fibers form hydrogen bonding and possible covalent interactions with the polar functional groups of epoxy, while alkali treatment of fibers further improves surface roughness and mechanical interlocking. In addition, CNTs with high surface area act as nanoscale bridges at the fiber-matrix interface, and when functionalized, they form strong physical and chemical interactions with the epoxy network. This synergistic effect of sisal fibers and CNTs results in improved load transfer efficiency and enhanced interfacial bonding.

**Fig. 8:**
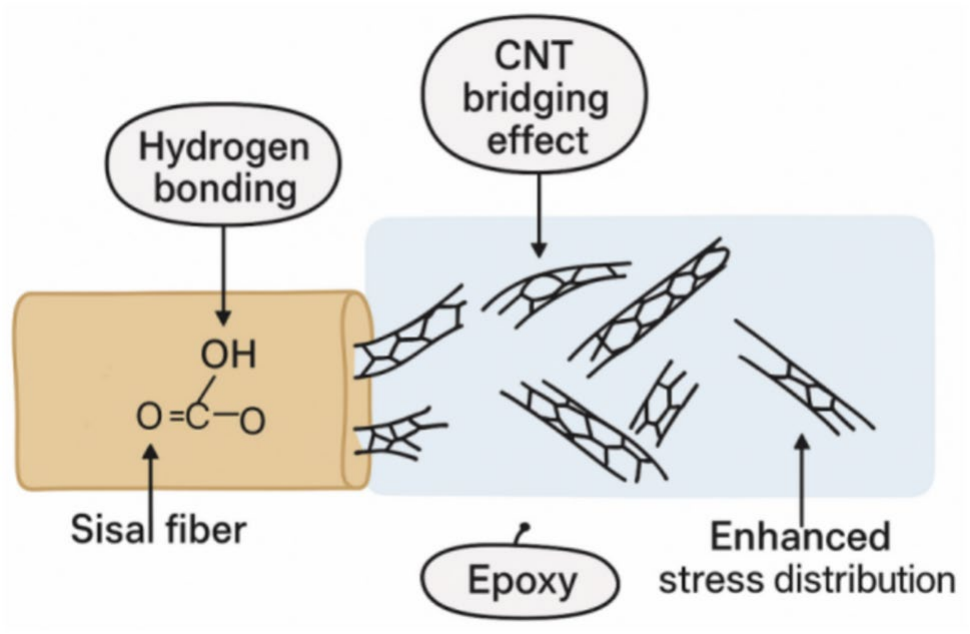
CNT assisted interfacial adhesion mechanism in sisal fiber-epoxy composites.

A conceptual schematic diagram is depicted in Fig. 8, illustrating how CNTs contribute to interfacial adhesion in sisal/epoxy composites. The Fig. 8 highlights the key mechanism including improved load transfer from matrix to fibers, CNT bridging at the interface, crack deflection, and the formation of a stronger interphase region. The Fig. 8 complements the SEM results by providing a clear representation of the reinforcing role of CNTs in enhancing the mechanical and tribological performance of the composites.

## Conclusions

The following conclusions may be drawn from the present study on the thermal and dynamic mechanical analyses of epoxy based bio-composites reinforced with 15 wt.% sisal fiber and varying amounts of carbon nanotubes (CNTs):

- The thermal gravimetric analysis (TGA) reported that all bio-composites undergo three main stages of degradation, with the lowest weight loss and highest thermal stability observed in the 1.0 wt.% CNT composite. Notably, this formulation exhibited the most resistance to thermal decomposition in the range of 100 °C –380 °C, confirming the role of CNTs in enhancing the thermal resistance up to an optimal content level.
- The differential scanning calorimetry (DSC) thermograms showed a pronounced endothermic peak at 110 °C, corresponding to moisture loss, particularly in composites with hydrophilic constituents such as cellulose and lignin from sisal fibers. Composites with 1.0 wt.% CNT showed increased crystallinity, leading to enhanced thermal stability.
- The storage modulus (E′) was maximized at 1.0 wt.% CNT with 3859.28 MPa, indicating optimal reinforcement and improved load bearing capacity due to effective dispersion and interaction of CNTs within the matrix.
- Composites with 1.0 wt.% CNT exhibited the highest loss modulus and the lowest damping factor ‘tan δ’, indicating both superior stiffness and reduced energy dissipation, the favorable traits for structural applications.
- The peak glass transition temperature (T_g_) of 89.26 °C in the 1.0 wt.% CNT composite, signifies increased thermal and dimensional stability.
- The surface morphology of bio-composites revealed that the optimal dispersion and strong interfacial bonding are achieved at 1.0 wt% CNT.

In summary, the incorporation of 1.0 wt.% carbon nanotubes into sisal fiber reinforced epoxy composite provides the most balanced enhancement in thermal stability, stiffness, and damping behavior, making it an optimal formulation for advanced bio-composite applications in automotive, construction, and packaging industries.

## Data Availability

The data presented in this study are available within the article.
